# Single-Heuristic Reasoning: Is It Still Dual-Process?

**DOI:** 10.3390/jintelligence11020033

**Published:** 2023-02-08

**Authors:** Pavle Valerjev, Marin Dujmović

**Affiliations:** 1 Department of Psychology, University of Zadar, 23000 Zadar, Croatia; 2 School of Psychological Science, University of Bristol, Bristol BS8 1QU, UK

**Keywords:** dual-process theory, reasoning, meta-reasoning, heuristics, intuitive reasoning, single-process reasoning, conflict detection, logical intuitions

## Abstract

Traditionally, paradigms used to study conflict in reasoning (and metacognition during reasoning) pit heuristic processes against analytical processes. Findings indicate that the presence of conflict between processes prolongs reasoning and decreases accuracy and confidence. In this study, we aimed to explore reasoning and metacognition when only one type of heuristic process is exploited to cue multiple responses. In two experiments, a novel modification of the Base Rate neglect task was used to create versions in which one belief-based heuristic competes, or works in concert, with another of the same type to provide a response. Experiment 1 results reveal that the presence of conflict between cued responses does not affect meta-reasoning, which indicates that reasoning defaulted to a single process. An alternative explanation was that the effect of conflict was masked due to an imbalance in the strength of the dominant response between conflicting and congruent versions. Experiment 2 was designed to test hypotheses based on these competing explanations. Findings show that when the strength of a response was no longer masking the effect, the conflict did result in longer reasoning times and lower confidence. The study provides more robust evidence in favor of the dual-process account of reasoning, introduces a new methodological approach, and discusses how conflict may be modulated during reasoning.

## 1. Introduction

Research into reasoning processes has been dominated by the dual-processing approach and the various theories it has spawned over the decades. Traditionally, System 1 or Type 1 processes were considered quick, automatic, cognitively undemanding, and based on intuitions. On the other hand, System 2 or Type 2 processes were considered slow, deliberate, cognitively demanding, and based on analytical thinking (Evans 2012). However, developments in the past two decades have led to the conclusion that responses previously ascribed to the slow System 2 are indeed available to System 1. Key findings leading to these conclusions come from research on reasoning under cognitive load and/or time pressure and research with the rethinking paradigm. When forcing participants to reason under cognitive load and under time pressure conditions, the frequency of analytical responses does decrease but only slightly, meaning that both intuitive and analytical responses are still being generated (Bago and De Neys 2017; Lawson et al. 2020). Similarly, when participants give quick initial responses and are then given the opportunity to rethink their choices, the vast majority of analytical responses after rethinking are not a result of switching from an intuitive response (Thompson et al. 2011; Dujmović et al. 2021). Rather, analytical responses after rethinking were already given as the initial response. These and other findings have led to a continuous development of models within the dual-process approach, which takes into account the fact that System 1 generates various types of responses, presumably accommodating different types of quick processes. An early idea about multiple intuitive processes is introduced by Glockner and Witteman (2010), while De Neys (2012) refers to logical intuitions which are part of System 1 and cue analytically correct responses in reasoning tasks. Logical intuitions would include mathematical, probabilistic, formal logic, and others not traditionally considered to be intuitions. The more traditionally used intuitions, like biases stemming from representativeness, matching, and other heuristics, will be referred to as *heuristic* intuitions.

**Figure 1:**
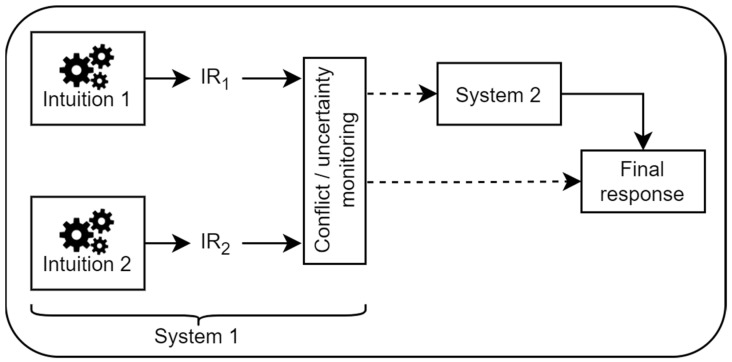
Schematic of a dual-process model of reasoning.

Based on these more recent findings, a common framework is emerging, and the proposed working model is depicted in Figure 1. Intuitive processes generate initial intuitive responses (IR). Depending on task demands, and previous experience, one or more of these responses may be generated. Most tasks in the field are designed to pit a heuristic response against an analytical response. For example, take a simplified version of the Base Rate Neglect task (De Neys and Glumicic 2008; Dujmović and Valerjev 2018). Participants are told that a random person is chosen from a group of 1000 people. The random person is very tall. The group consists of 10 basketball players and 990 mathematicians. The task is to pick whether it is more probable that the randomly chosen person is a basketball player or a mathematician. In this example, one intuitive response is based on height being extremely typical of basketball players compared to mathematicians. Thus, it indicates that the randomly chosen person is most likely a basketball player. The second response is based on the information indicating there are many more mathematicians in the group, and thus indicates that the randomly chosen person is a mathematician.

The two initial responses in this example are conflicting, but responses could point towards the same option congruently. The strength of these initial intuitive responses is going to depend on a number of factors. Stanovich (2018) refers to *mindware* as one determining factor. Mindware refers to knowledge and experiences of rules, procedures, and strategies which help detect and override intuitive, miserly, but incorrect responses. In traditional heuristic and bias tasks, the mindware necessary to perform well is usually tied to probabilistic, causal, and scientific reasoning as well as numeracy. The heuristic that basketball players are very tall has been built through experience and many examples over a lifetime. The same can be said about relying on the high proportion of mathematicians in the group. Knowledge and experiences handling various quantities, ratios, and probabilities build up mindware which can intuitively use that type of information. The strength of each intuitive response will therefore depend on the instantiation of the mindware. The average person might have a much stronger mindware generating the heuristic response (basketball player) when compared to a professional statistician who may have built up a strong mindware for handling probabilities, which favors what is deemed to be the analytical response (mathematician). De Neys (2022) further speculates that the strength of these intuitions might be variable over time. Therefore, the relative strength of intuitive responses may not simply be a function of peak magnitude but also the point in time at which the system is prompted to give a response. In our previous work, we have shown that the relative strength of responses can be influenced via instruction (Valerjev and Dujmović 2017) as well as a change in the modality of presentation (Dujmović and Valerjev 2017), implying attention and attentional resources play a mediating role between mindware and response strength. When the two responses are congruent, no further deliberation or activation of System 2 is required since there is no uncertainty, and a final response is given. If the responses are in conflict, a monitoring process may trigger System 2 activation. It is not clear how exactly conflict or the resulting uncertainty is computed, but many studies have shown that the presence of conflict increases the likelihood of System 2 activation (Dujmović and Valerjev 2018; Pennycook et al. 2014, 2015; Thompson and Johnson 2014). When the level of uncertainty/conflict is high enough to be detected, System 2 is activated to resolve the situation. This may include rationalization of one of the responses, considering additional evidence for one or both responses, or attempting to generate a novel response. If the level of uncertainty does not trigger System 2, the final response is simply the stronger of the two initial responses.

In recent years, more researchers have recognized the importance of metacognitive processes during reasoning. Indeed, the processes that detect conflict and/or compute uncertainty could be viewed as metacognitive monitoring. This has led to the emergence of meta-reasoning (Ackerman and Thompson 2015, 2017), in which measuring various metacognitive judgments such as perceived difficulty, solvability, and confidence has accompanied the usual measures of accuracy (usually defined as the proportion of analytical responses) and response time. In fact, metacognitive measures have proven to be very useful and sensitive to experimental manipulations even when accuracy and response times are not. For example, if all conditions in a task are completed at a very high level of accuracy, then there will be very little or no difference among them, but confidence judgments (for example) may be impacted by the experimental manipulation. Standard findings show participants are less confident in conflict when compared to congruent versions of reasoning tasks (Dujmović and Valerjev 2018; Dujmović et al. 2021; Mevel et al. 2015; Shynkaruk and Thompson 2006). Accuracy is often not a good predictor of confidence (Bajšanski et al. 2014; Markovits et al. 2015; Thompson et al. 2011), while response time as a proxy for response fluency oftentimes is (Ackerman and Zalmanov 2012; Dujmović and Valerjev 2018; Thompson et al. 2013; Thompson and Johnson 2014).

While the introduction of metacognitive measures to reasoning tasks has increased our understanding of both metacognitive and reasoning processes, it is becoming increasingly more difficult to probe deeper into these processes using current research paradigms. The tasks which have been used in reasoning research track their roots to Tversky and Kahneman (1982, 1983) and their conjunction fallacy (Linda problem) task. The task describes Linda, an outspoken, former philosophy student who was concerned with issues of discrimination and social injustice and participated in a number of demonstrations. Participants are then asked whether, years later, it is more probable that A—Linda is a bank teller or that B—Linda is a bank teller *and* active in the feminist movement. In many different variations of this task, Tversky and Kahneman found that the vast majority of participants judged the conjunction of two statements (bank teller *and* active feminist) as more likely than one of the two constitutive statements (bank teller). This is a fallacy; the conjunction of any two events is less likely than either of the two individually. The fallacy is committed because the description of Linda is more representative of someone who is to become active in the feminist movement, and thus, the conjunction is deemed more probable than the single statement even though it is, in fact, less probable (for a thorough investigation of the conjunction fallacy from a modern meta-reasoning perspective see Dujmović et al. 2021). Considering how prevalent the fallacy was and how difficult it was for participants to give the correct response, it naturally served as evidence for reasoning being dual-process. The representativeness heuristic provided one response (the conjunction) and correct probability assessment the other. This type of design then became prevalent in practically all reasoning tasks of note. This includes the various versions of the Base Rate Neglect task (De Neys and Glumicic 2008; Dujmović and Valerjev 2018), the Cognitive Reflection Test (Frederick 2005), the Covariation Detection task (Stanovich et al. 2016; Valerjev and Dujmović 2019), and syllogisms (Shynkaruk and Thompson 2006). In all of these tasks, the design pits one type of process (e.g., representativeness or believability) against another (e.g., probability computation or formal logic) in conflict versions or has the two processes aim at the same response in congruent versions. While these tasks have produced many important findings, there are inherent limitations in how they have been used. Namely, the tasks always pit qualitatively different processes of unknown strengths against each other. One can vary the group sizes in a base rate task (Dujmović and Valerjev 2018), but the strength of the heuristic response is almost always unknown or only roughly estimated as more or less extreme. Likewise, one can vary the believability when using syllogisms (Shynkaruk and Thompson 2006). However, with any of these manipulations, the relative strengths of the two processes are unknown. Indeed, the fact that the mismatch is quite extreme in most cases has perpetuated what De Neys (2022) deems as an unfounded assumption of exclusivity (that some responses are only available to System 2). The mismatch can be seen as a severe difference in mindware. In the Linda problem, representativeness as a heuristic is well developed for the vast majority of people, while properly combining probabilities or understanding rules of formal logic is tied to a much weaker mindware for most. Even if one could match (via pre-studies) the strengths of one process with another, these processes are still qualitatively different, and it is unknown if a match in peak estimated strength means a match when embedded in a reasoning task. Without more stringent matching, subtle manipulations are more difficult, as is testing more nuanced aspects of dual-process models.

The aims of this study were two-fold. First, we aimed to develop materials for reasoning tasks that focus on only one type of heuristic process with good measurements of relative heuristic strength. In order to accomplish this, we started from the simplified Base Rate task, but instead of pitting a heuristic intuitive process against a logical intuitive process, we chose to use the same type of intuitive heuristic process in order to cue both responses. In comparison to the example given above, instead of stating that a randomly chosen, very tall person came from a group of 10 basketball players and 990 mathematicians, we would state that a randomly chosen person is very tall and very intelligent while being selected from a group which is half basketball players and half mathematicians. In this version, being very tall is indicative of being a basketball player, while being very intelligent is indicative of being a mathematician (for details on all conditions and materials, see the Methods section of Experiment 1). Second, we aimed to test whether standard findings considering the influence of conflict detection and resolution hold for these tasks in which the same type of intuitive process cues different responses. Our pre-study (see Methods) results made fine control of the level of conflict/congruence possible. This allows for comparisons of congruent and conflict conditions in which the dominance of the final response can be held constant, making an evaluation of the influence of conflict easier. Given previous research, we hypothesized that conflict would manifest through longer reasoning times, lower accuracy (measured as a lower proportion of response in accordance with the dominant heuristic), and lower confidence. It was further hypothesized that the level of congruence/conflict would influence reasoning speed, accuracy, and confidence. Higher congruence should result in an increase in speed, accuracy, and confidence, while higher conflict should result in a decrease in all three dependent variables.

## 2. Experiment 1

### 2.1. Methods

In order to grasp the design of Experiment 1 and the materials created for this purpose, it is important to first explain the principles and the pre-study based on which these materials were created. In the first phase of the pre-study, participants (*N* = 22) generated typical and atypical characteristics for each of the 12 groups of people (e.g., mathematicians, basketball players, etc.). Based on the materials generated, 12 characteristics were chosen for the second phase in which participants (*N* = 309) judged the typicality of each characteristic for each of the groups on a –4 to 4 scale where high scores indicate very typical characteristics, while low scores indicate very atypical characteristics (e.g., being very tall is very typical of basketball players, but being very wealthy is atypical of cleaning ladies). These typicality judgments can then be used in various combinations in the manner depicted in Table 1. The heuristic response based on a characteristic will depend on the relative typicality of the characteristic for either of the two groups. In Table 1, the first characteristic (e.g., being highly educated) is quite typical of group A (e.g., mathematicians) but not group B (e.g., basketball players). The resulting heuristic response is that the person is more likely to be from group A, with a strength of 3.45. The reverse pattern is true for the second characteristic (e.g., being physically strong), which is typical of group B (basketball players) but not of group A (mathematicians). For this characteristic, the person is more likely to be from group B with a strength of 2.

These two heuristics could be combined in a single task if the objective was to select to which group a randomly chosen person high on both characteristics (highly educated and physically strong) belongs. Table 1 depicts an example of conflict, where one heuristic process results in one strong response, whereas the other heuristic process results in a competing response. These tasks can be designed for congruent as well as conflict conditions. In the congruent condition, both heuristic processes indicate the same response; therefore, the nominal strength of the probable final response is the sum of the two heuristic response strengths. Alternatively, in the conflict condition, the final response strength is the difference between the two heuristic response strengths. This also allows us to vary the level of congruence and conflict since the magnitude of the final response strength is a function of these levels. It is worth noting that only the typicality values from Table 1 were measured, while combining them to compute all other values served to produce the stimuli for our experimental conditions. If a similar computation does occur in the brain, it is likely to be considerably more complex and include additional factors.

#### 2.1.1. Design

As mentioned, based on typicality judgments, tasks with multiple characteristics can be formed such that heuristic processes are congruent or in conflict, and the level of each can be varied. Experiment 1 follows a 2 (congruence) × 2 (level) design. Tasks could be congruent or in conflict, and the level of congruence/conflict had two magnitudes (low and high). In the low-level conditions, the final response strength was (on average) 1.50. Therefore, the low-congruent and low-conflict conditions were particularly interesting since the final response strength remained identical. In the high-level conditions—the high conflict was achieved by combining heuristic responses of equal nominal strengths but opposite directions, resulting in a final response strength of 0. This is the maximal level of conflict/uncertainty. In the high-congruence condition, two strong heuristics indicating the same response were combined to form tasks with an average nominal strength of 5.

#### 2.1.2. Participants

A total of *N* = 145 participants were recruited amongst undergraduate students across a number of Croatian universities. After processing, the data from *N* = 139 participants (92.70% female, *M*_age_ = 22.28, *SD*_age_ = 3.31) was retained for statistical analyses. Sensitivity analysis conducted in G*Power v3.1.9.4 reveals that with a sample of *N* = 139, a desired power level of .80, and an alpha of .05, the smallest effect we could confidently detect was η_p_^2^ = .054.

#### 2.1.3. Materials

A total of 16 tasks were generated (four per condition) by combining typicality scores into heuristic response strengths resulting in different combinations of congruence and level. Table 2 describes all four conditions with examples. Each task consisted of a scenario in which a person was selected at random from a group. Half of the group were members of one cohort (e.g., scientists) and half of another (e.g., gymnasts). The randomly chosen person is described with two characteristics (e.g., very intelligent and physically strong). The question posed to participants was whether it was more probable that the randomly chosen person was a member of one cohort or the other.

In the pre-study, typicality judgments were made independently for each group rather than relatively across groups. Therefore, groups in all tasks were made up of cohorts of the same gender (e.g., ballerinas and cleaning ladies). This was done in order to control for the fact that typicality judgments were likely given in relation to other same-gender individuals. For example, physical strength was judged as highly typical for both male basketball players and female gymnasts. If these groups were part of the same trial, there is no doubt that the characteristic would be associated with basketball players to a much higher degree than gymnasts, even though the typicality judgments would not suggest it.

#### 2.1.4. Procedure and Data Processing

**Figure 2:**
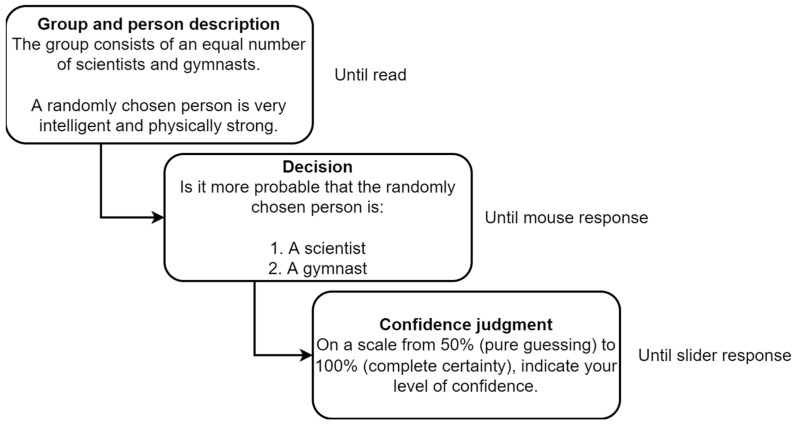
Single-trial procedure for tasks in Experiment 1.

Participants first provided demographic information and were given instruction in the form of a walkthrough of an unused example of the task. They then completed the 16 tasks (a single trial procedure for each task can be seen in Figure 2). The task order was randomized for each participant. The order in which responses appeared was balanced so that in half of the trials, the response according to the dominant heuristic was the first response option, and in half, it was the second option. The experiment was designed in PsychoPy (Peirce et al. 2019) and conducted via the Pavlovia online platform. Participants were required to complete the study on a desktop or laptop computer with a mouse and asked to isolate themselves in a quiet environment during the study.

Reading times in the Group and personal information, response times in the Decision phase, and accuracy and confidence judgments were recorded for each trial. Responses were considered as correct if they were in line with the more dominant heuristic (according to Table 2). The high-conflict condition was an exception since there was no dominant response; as such, the top response to appear in the Decision phase was coded as correct for convenience. Considering that the reasoning process included both the first and second phases (Figure 2), the total time for each trial was computed as a sum of reading and decision time.

Only trials with correct responses were selected for further analyses of reasoning times and confidence judgments (but all trials in the high-conflict condition). This was done because correct trials made up the vast majority of all trials and because a response in line with the probable final response according to our nominal strengths indicates that it did indeed fit within the ascribed experimental condition for the particular participant. Additionally, we excluded 5% of all trials based on total time (2.5% of the fastest and 2.5% of the slowest), considering this was an online study, and these more extreme times deviated considerably from the median. After this filter was applied, a median for each of the dependent variables was computed for each participant in each experimental condition to form the final dataset for analysis.

### 2.2. Results and Discussion

Total times were considerably positively skewed; therefore, analyses were conducted after computing the natural logarithm, and no other data processing was required prior to running further analyses. In order to determine whether congruence and level had an effect on accuracy, reasoning times, and confidence judgments, three 2 × 2 repeated measures ANOVAs were conducted (results can be seen in Table 3).

**Figure 3:**
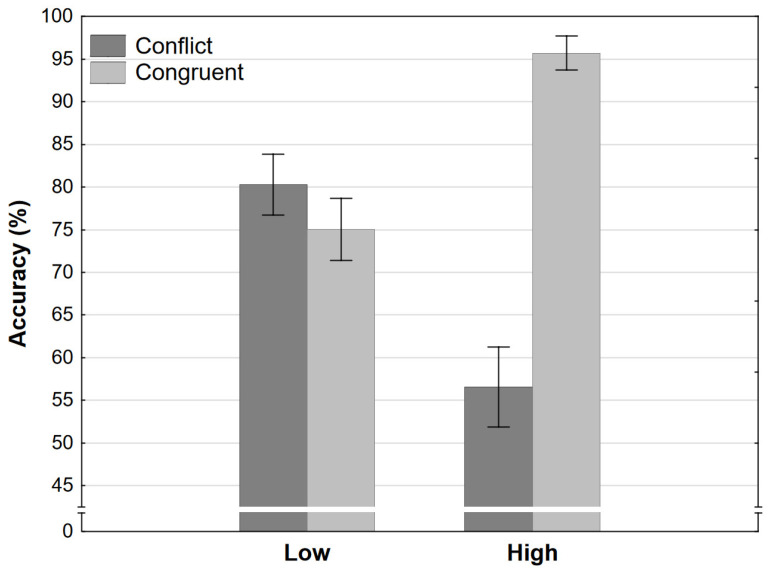
Accuracy as a function of congruence and level in Experiment 1 (error bars represent 95% CI).

The ANOVA of accuracy rates revealed that participants were more accurate in congruent conditions. However, this is qualified by a large interaction effect. Participants were equally accurate in low-conflict and low-congruent conditions (as tested by Tukey HSD post hoc tests) while being significantly more accurate in the high-congruence condition and significantly less accurate in the high-conflict condition (Figure 3). Note that accuracy in the high-conflict condition refers to the percentage of times participants chose the first response which appeared in the trial. If our tasks were perfectly calibrated for every single participant at a nominal strength of 0, we would expect the ratio to be exactly 50%. As can be seen in Figure 3, accuracy was only slightly above 50%, meaning that when the conflict was very high, participants chose randomly most of the time with a slight preference for the first response presented in the Decision phase (Figure 2). This may be an interesting effect of primacy. Both of the responses are cued strongly by opposing processes, and the first one may have a slight advantage simply due to primacy. The more important result in the context of our aims is that participants were not significantly more accurate in both of the congruent conditions when compared to conflict conditions. In fact, participants were slightly (though not significantly) more accurate in the low-conflict when compared to the low-congruence condition. This implies that the nominal strength of the final response itself was the determining factor rather than whether conflict was present or not.

**Figure 4:**
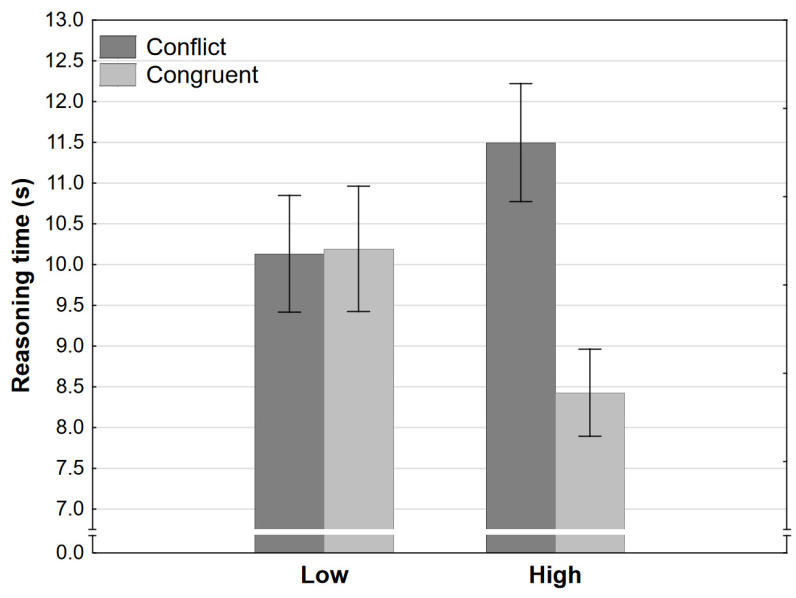
Reasoning time as a function of congruence and level in Experiment 1 (error bars represent 95% CI).

The ANOVA of reasoning times revealed the same pattern of results. Participants were generally faster in the congruent conditions, but this is qualified by a large interaction effect. Participants completed tasks in the low-conflict condition just as fast as in the low-congruence condition but significantly faster in the high-congruence and significantly slower in the high-conflict condition (Figure 4, note that the figure depicts raw response times for convenience, the ANOVA was conducted on logarithms). The implications are the same as for accuracy rates; it seems that final response strength is the key determining factor in how long reasoning is going to take, not whether conflict was present or not.

**Figure 5:**
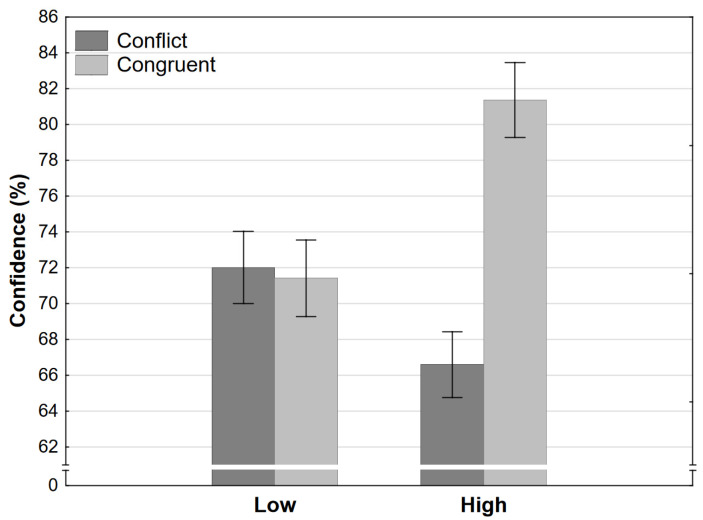
Confidence judgments as a function of congruence and level in Experiment 1 (error bars represent 95% CI).

Finally, the ANOVA of confidence judgments resulted in the same pattern. The large interaction effect was significant because there was no difference between the low-level conditions, while participants were much more confident in the high-congruence and much less confident in the high-conflict condition (Figure 5).

These results would indicate, contrary to modern dual-processing theories, when two heuristic processes of the same type are pitted against each other, the presence of conflict does not impact reasoning. The key comparison which leads to this conclusion is between the low-conflict and low-congruence conditions. If both conflict and nominal final response strength were influencing reasoning, given that strength is equal for these two conditions, participants should have been more accurate, faster, and more confident in the low-congruence condition. On the other hand, if only strength influenced reasoning, then we would expect exactly the pattern of results we observed in Experiment 1. Note that the remaining two conditions would result in the exact same pattern of results regardless of the explanation. The result seems to imply that when a single type of heuristic process is required to cue responses, only one heuristic process actually *is* activated. This heuristic process would then compute all of the information of the same type and cue one of the responses with some level of certainty.

However, the low-congruent and conflict conditions are not entirely balanced, nor can complete balance be achieved due to the fact that one combines the strengths of two responses while the other is a difference between them. Table 4 highlights this difference.

Apart from final response strength and the presence of conflict, there is a third difference between the conditions which may influence reasoning. Note that the strength of heuristic responses in the congruent condition is quite low, even though they both cue the same response. On the other hand, even though competing responses are cued in the conflict condition, the dominant response is very strong. This high level of strength, not present for either heuristic response in the congruent condition, may mitigate the effect of conflict on reasoning. The relative dominance of one response might make conflict detection less probable, or it may make it easier to resolve conflict in favor of the more dominant heuristic response. The final response then may seem more salient to participants. These effects may contribute to the low-conflict condition resulting in the same accuracy, speed, and confidence as the low-congruent condition. It has to be noted again that the nominal strengths of final responses and how they are computed are surely an oversimplification of any such computation during reasoning. Regardless of whether and how this computation is done—it may be modulated by the strength of cued heuristic responses. While the final response strength is nominally +1.50 in both situations, that strength is computed based on independent typicality judgments and then combined with the estimated strengths of heuristic responses. During reasoning, the presence of conflict (and how large the conflict is) would modulate final response strength to a lower score, but the relative dominance of one heuristic response would increase the score. In the congruent condition from Experiment 1, the reverse may be true, congruence (or absence of conflict) would strengthen the final response strength, but lack of a strong heuristic response would decrease (or at least not contribute as much) to the score. Thus, the effect of conflict, indirectly measured by looking at the differences between these two conditions, may be masked.

## 3. Experiment 2

It is not possible to completely balance congruent and conflict conditions. Nominal strengths of final responses can be kept identical, as in the low-conflict and low-congruence conditions in Experiment 1, but simply due to the nature of the conflict condition having competing heuristics pulling in opposite directions, it, by definition, means that the stronger heuristic in the conflict condition will be stronger than either of the heuristic responses in the congruent condition. However, nominal heuristic response strength likely would not have a linear, unbound effect. The logic behind Experiment 2 was to increase the strength of heuristic responses in both the congruent and conflict conditions sufficiently so that the difference between them would not mask the effect of conflict if it does influence reasoning. Therefore, the aim of Experiment 2 was to resolve which of the two explanations led to the pattern of results in Experiment 1.

### 3.1. Methods

#### 3.1.1. Design

The design of Experiment 2 was quite similar to Experiment 1. It was again a 2(congruence) × 2(level) design. The main difference is that instead of the *level* independent variable relating to low or high congruence, and low or high conflict, it now directly relates to the nominal strength of the final response. This strength in the low conditions was again +1.50, which allows for the replication of the main, unexpected finding from Experiment 1. The strength in the high conditions was, on average, +3.00 for both congruent and conflict conditions. If strength was the only influence on reasoning, then we expected just a main effect of strength level when analyzing accuracy, reasoning times, and confidence. Increasing strength should lead to higher accuracy, faster reasoning, and higher confidence, irrespective of congruence. If, on the other hand, the presence of conflict does influence reasoning, then we would expect a different pattern. Given the results from Experiment 1, we would expect a congruence by strength-level interaction effect. The increase in accuracy, speed up of reasoning, and increase of confidence at higher nominal strength should be significantly larger in the congruent when compared to the conflict condition.

#### 3.1.2. Participants

A total of *N* = 117 participants were recruited amongst undergraduate students across a number of Croatian universities. After data processing, a total of *N* = 114 participants (86.73% female, *M*_age_ = 22.78, *SD*_age_ = 6.84) were retained for statistical analyses. Sensitivity analysis conducted in G*Power v3.1.9.4 reveals that with a sample of *N* = 114, a desired power level of .80, and an alpha of .05, the smallest effect we could confidently detect was η_p_^2^ = .065.

#### 3.1.3. Materials

The materials for Experiment 2 were constructed using similar logic. In fact, the same types of tasks from the low-conflict and congruent conditions from Experiment 1 (Table 2) were used in Experiment 2. New types of tasks were created for high-strength conflict and congruent conditions.

As can be seen in Table 5, the strength of the final response was equalized across these two conditions, but unlike the low-congruence condition from Experiment 1, the high-strength congruent condition here had an increased level of strength for both heuristic responses, as discussed in the Design section. The precautions taken when generating the tasks and the number of tasks per condition were the same as in Experiment 1.

#### 3.1.4. Procedure and Data Processing

The procedure and all data pre-processing mirrored Experiment 1.

### 3.2. Results and Discussion

Natural logarithms of response times were again computed for further analyses, and no other data processing was required. In order to test whether congruence and strength level had an effect on reasoning, three 2 × 2 ANOVAs were conducted on the accuracy, reasoning times, and confidence judgments (results in Table 6).

**Figure 6:**
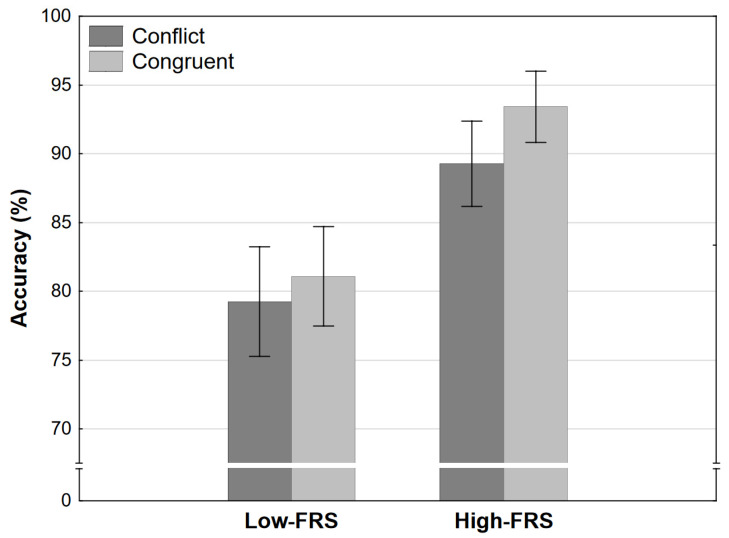
Accuracy as a function of congruence and final response strength level in Experiment 2 (error bars represent 95% CI). FRS refers to the final response strength level (see Table 1).

The analysis of accuracy revealed a strong effect of strength level and no other significant effects. Participants were more accurate (chose the dominant response at a higher rate) in high-strength conditions irrespective of congruence (Figure 6). There was a slight trend towards higher accuracy in congruent conditions in general, but this did not reach significance.

**Figure 7:**
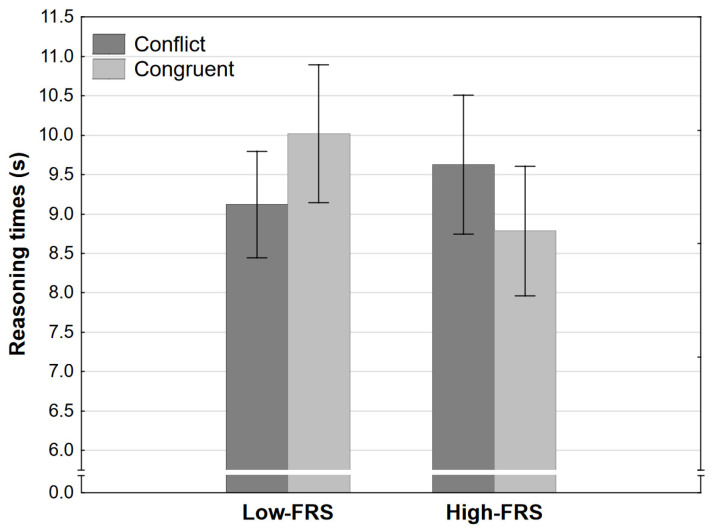
Reasoning time as a function of congruence and final response strength level in Experiment 2 (error bars represent 95% CI). FRS refers to the final response strength level (see Table 1).

Reasoning time analysis, however, did result in a significant interaction effect. The increase in nominal strength led to a significant decrease in reasoning times in the congruent but not in the conflict condition (Figure 7). The main effect of the level was also significant but inconsequential considering the interaction effect and the fact that effect size was under what our sensitivity analysis indicates would be the smallest confidently detectable effect.

**Figure 8:**
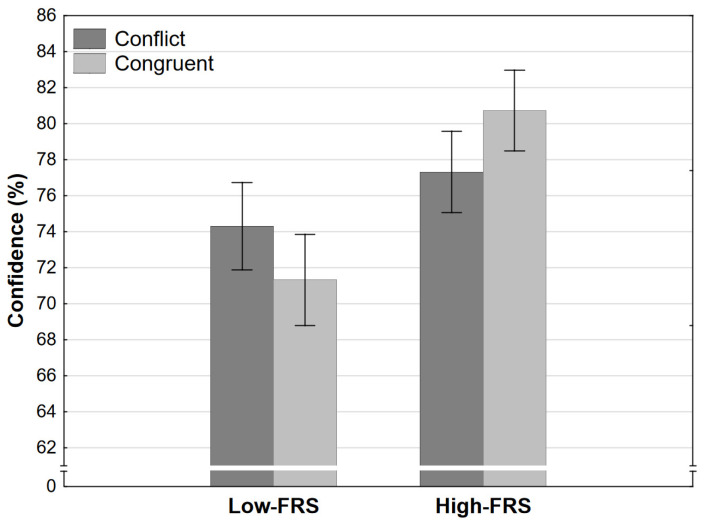
Confidence as a function of congruence and final response strength level in Experiment 2 (error bars represent 95% CI). FRS refers to the final response strength level (see Table 1).

Finally, the analysis of confidence judgments revealed both a strong effect of strength level and a strong interaction effect. Participants were generally more confident when reasoning in high-strength conditions (Figure 8). However, the increase of confidence between high and low strength levels was significantly larger in the congruent than it was in the conflict condition.

Overall, results from Experiment 2 replicate the finding of Experiment 1 in the low-level conditions and provide evidence that conflict does affect reasoning performance. This effect seems to have been masked in Experiment 1 due to the strength of the dominant heuristic, but the masking was reduced in Experiment 2, revealing the effect of conflict. The increase of the prevailing response strength had a larger impact on reasoning in the congruent than it did in the conflict condition (a decrease in reasoning times and a larger increase in confidence).

## 4. General Discussion

We set out to test whether conflict detection and resolution retained a strong influence on meta-reasoning when pitting responses cued by the same type of heuristic process rather than two vastly different processes. Thus far, the influence of conflict in reasoning has always been tested by inducing processes relying on different types of mindware. Further, the strength of responses cued by these processes is not strongly controlled or calibrated. Usually, the only manipulation is whether a conflict is present or not; at the most, the level of conflict is lightly manipulated when it is simple to do so (e.g., ratios in the Base Rate task as in Dujmović and Valerjev 2018). Therefore, it is difficult to disambiguate the influence of the presence and magnitude of the conflict itself from other differences between the processes cueing these responses. The key finding from Experiment 1 was that when nominal strengths of the prevailing responses were matched in congruent and conflict conditions, there was no difference in accuracy, reasoning times, or confidence. These findings are in sharp contrast with most research within the modern dual-process framework (Dujmović and Valerjev 2018; Dujmović et al. 2021; Mevel et al. 2015; Shynkaruk and Thompson 2006). Indeed, the most parsimonious explanation of the results was a single-process account in which one heuristic process simply computes all of the information in the task and cues a response with some level of certainty. However, as pointed out in the discussion of Experiment 1, the presence or absence of conflict was not the only difference between the two key conditions. The strength of the dominant heuristic response was also different, which is unavoidable due to the nature of conflict vs. congruent tasks if one aims to match the nominal strength of final responses. This may have masked the impact of conflict in Experiment 1. In order to account for this, Experiment 2 was designed to test whether an increased nominal strength level would have more of an impact on congruent conditions than conflict conditions. We hypothesized that an increase in heuristic response strength would, even if the two conditions remained unbalanced in that regard, unmask the effect of conflict if there was one. Results indeed revealed that increased strength had a higher impact in the congruent condition. Accuracy was equally increased between strength levels irrespective of congruence, but analyses of the remaining two dependent variables revealed strong interaction effects. Participants reasoned significantly faster in the high-strength congruent when compared to the low-strength congruent condition, while the difference between the two conflict conditions was not significant. Further, confidence was higher with the increase of strength level in both congruent and conflict conditions, but the increase was significantly larger in the congruent condition. The results indicate that even when a single type of heuristic process is responsible for cueing multiple responses, there is an effect of conflict. This provides further evidence that two heuristic processes cue the responses; conflict can then be detected and does influence meta-reasoning.

The results revealed an interesting modulation effect of dominant response strength on the impact of conflict in our tasks. A higher strength of the dominant response can mitigate the negative impact conflict has on reasoning times and confidence levels. This may be due to factors that are not mutually exclusive. First, there may be a lower probability of conflict being detected due to the high strength of one of the responses. Second, it may be due to an easier resolution of the conflict when it is detected. It is likely that this effect is bound and non-linear. In Experiment 2, the high-strength conflict condition boasts a higher final response strength (which can be seen as lower conflict), a higher heuristic strength of the dominant, and a lower heuristic strength of the subordinate heuristic response than the low-strength conflict condition. However, these conditions do not differ when looking at reasoning times. Additionally, the increase in confidence was much smaller than in the congruent conditions—despite both heuristic responses in the high-strength congruent condition being much weaker than the dominant response in the high-strength conflict condition.

The findings presented here provide a promising foundation as the approach taken allows for more control and a more detailed analysis of the targeted processes. As far as we are aware, this is the first study to investigate processing that gives rise to System 1 responses rather than just the outcomes of those processes. In the future, materials can be generated using a similar approach in order to match the strength of qualitatively different heuristic processes and explore differences due to other factors. The recent review of dual-process theory by De Neys (2022) poses questions for future research, many of which we feel our approach is uniquely positioned to explore. That being said, there are clear limitations to this approach. There is a clear disconnect between independent typicality judgments and solving reasoning tasks with combinations based on those judgments. Indeed, additional factors that occur during reasoning led to Experiment 1 findings fitting a misleading single-process account. Furthermore, it is entirely unknown whether computations similar to the ones used here to generate experimental conditions mirror anything happening in the brain during reasoning. If they do, they are likely to be an oversimplification of the actual computations. However, the behavioral results presented here match expectations based on our computations rather well (e.g., participants basically guessing in the high-conflict condition of Experiment 1), and this approach may be an avenue to better explore the dynamics of reasoning in the future.

## Figures and Tables

**Table 1:** Schematic example of combining characteristics to form conditions.

	Typicality		Probable Heuristic Response (HR)	Nominal Strength of HR ^1^	Probable Final Response (FR)	Nominal Strength of FR ^2^
	Group A	Group B				
Characteristic 1	3.50	0.05	A	3.45	A	1.45
Characteristic 2	0.50	2.50	B	2.00		

^1^ The difference in typicality scores; ^2^ The difference of HR strengths in conflict, and the sum of strengths in congruent conditions (in the direction of the dominant response if there is one).

**Table 2:** Examples from all experimental conditions in Experiment 1.

	High		Low	
	Conflict	Congruent	Conflict	Congruent
Group 1	Scientists	Ballerinas	Actresses	Boxers
Group 2	Gymnasts	Cleaning ladies	Scientists	Basketball players
Characteristic 1 (strength of HR)	Intelligence (+2.40 group 1)	Gentleness (+2.50 group 1)	Sociability (+2.80 group 1)	Intelligence (+0.75 group 2)
Characteristic 2 (strength of HR)	Strength (+2.40 group 2)	Beauty (+2.50 group 1)	Diligence (+1.30 group 2)	Wealth (+0.75 group 2)
Strength of FR	0	+5 group 1	+1.50 group 1	+1.50 group 2

Note: HR—Heuristic response; FR—Final response.

**Table 3:** Congruence by level ANOVA results for accuracy, reasoning times, and confidence in Experiment 1.

Effect	Accuracy			Reasoning Times			Confidence		
	*F*(1, 138)	η_p_^2^	*p*	*F*(1, 138)	η_p_^2^	*p*	*F*(1, 138)	η_p_^2^	*p*
Congruence	93.82	.41	<.001	48.77	.26	<.001	112.51	.45	<.001
Level	0.72	<.01	.399	0.62	<.01	.431	18.46	.12	<.001
Interaction	193.38	.58	<.001	36.91	.21	<.001	182.39	.57	<.001

**Table 4:** Schematic of differences between low congruent and conflict conditions.

	Congruent	Conflict
Strength of HR_1_	+0.75—Group A	+3.00—Group A
Strength of HR_2_	+0.75—Group A	+1.50—Group B
Strength of FR	+1.50—Group A	+1.50—Group A

Note: HR—Heuristic response; FR—Final response.

**Table 5:** High-strength conflict and congruent conditions in Experiment 2.

	Conflict	Congruent
Group 1	Actresses	Scientists
Group 2	Cleaning ladies	Ballerinas
Characteristic 1 (strength of HR)	Wealth (+4.00 group 1)	Strength (+1.50 group 2)
Characteristic 2 (strength of HR)	Strength (+1.00 group 2)	Dexterity (+1.50 group 2)
Strength of FR	+3.00 group 1	+3.00 group 2

Note: HR—Heuristic response; FR—Final response.

**Table 6:** Congruence by response strength level ANOVA results for accuracy, reasoning times, and confidence in Experiment 2.

Effect	Accuracy			Reasoning Times			Confidence		
	*F*(1, 113)	η_p_^2^	*p*	*F*(1, 113)	η_p_^2^	*p*	*F*(1, 113)	η_p_^2^	*p*
Congruence	3.86	.03	.052	0.16	<.01	.687	0.10	<.01	.752
Level	45.53	.29	<.001	4.55	.04	.035	64.83	.36	<.001
Interaction	0.51	<.01	.477	10.35	.08	.002	16.81	.13	<.001

## Data Availability

The data presented in this study are openly available on the Open Science Framework at https://doi.org/10.17605/OSF.IO/J8XTN.
